# Les fistules œsotrachéales congénitales isolées à propos de 2 cas

**DOI:** 10.11604/pamj.2014.17.304.3958

**Published:** 2014-04-22

**Authors:** Imad El Biache, Maryem Lechqar, Mohammed Rami, Youssef Bouabdallah

**Affiliations:** 1 Chirurgie Pédiatrique, Centre Hospitalier Universitaire Hassan II, Fès, Maroc

**Keywords:** Fistule œso-trachéale isolée, Transit oesophagien, cervicotomie, isolated congenital tracheoesophageal, esophageal transit, cervicotomy

## Abstract

Les auteurs rapportent 2 cas de fistules oesotrachéales isolées sans atrésie de l'oesophage, colligés au service de chirurgie pédiatrique au CHU Hassan II de Fès au Maroc entre 2008 et 2013. Il s'agit d'une anomalie rare représentée par un fin canal ascendant entre l'oesophage et la face postérieure de la trachée, à la hauteur du défilé cervico-thoracique. Elle se manifeste cliniquement par une symptomatologie respiratoire parfois digestive. Le diagnostic a été confirmé par le transit oesophagien dans les 2 cas et a permis aussi de déterminer le siège de la fistule. Le traitement était chirurgical, il a permis de supprimer la communication anormale entre l'oesophage et la trachée par un abord cervical avec interposition musculaire dans les 2 cas. Les suites post-opératoires et l’évolution à long terme étaient simples. Le but de ce travail est d'exposer les différents moyens diagnostique et thérapeutique.

## Introduction

Une fistule oesotrachéale peut exister sans atrésie de l'oesophage, mais cette forme ne représente que 4% des anomalies congénitales de l'oesophage [1]. Une fois le diagnostic confirmé, il faut rechercher les malformations associées notamment celles de l'appareil respiratoire qui sont assez fréquentes [2]. Le traitement est toujours chirurgical et doit être précoce pour un meilleur pronostic [3].

## Méthodes

### Observation 1

**Figure 1:**
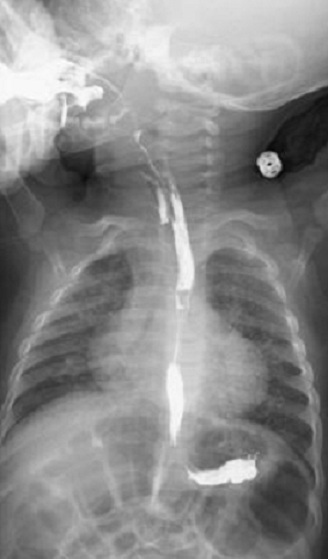
Transit œsophagien qui objective une fistule oeso-trachéale se projetant à hauteur de la première vertèbre dorsal

C.W, nourrisson de 14 mois, en cours de vaccination, hospitalisé pour broncho-pneumopathies récidivantes. Son histoire de la maladie remonte à la naissance par des fausses routes aggravées 2 semaines après par des épisodes de bronchites à répétition, traitées à maintes reprises par antibiothérapie et traitement symptomatique sans amélioration. L'enfant fut adressé en pédiatrie pour suspicion de reflux gastro-oesophagien où un TOGD a été réalisé montrant une FOT en regard de D2 (Figure 1). A l'admission, l'examen clinique a noté des râles ronflants bilatéraux à l'auscultation pulmonaire. La radiographie pulmonaire a visualisé une opacité lobaire moyenne droite. Une fibroscopie oesophagienne préopératoire a permis de repérer la fistule avec mise en place d'une sonde gastrique s'arrêtant au niveau de la fistule. La chirurgie s'effectue par cervicotomie droite avec suture sur l'oesophage sur sonde gastrique tutrice et mise en place d'un patch musculo-aponévrotique entre la trachée et l'oesophage, les suites postopératoires étaient simples, un TOGD de contrôle a 1 mois montre l'absence de trajet fistuleux.

### Observation 2

**Figure 2:**
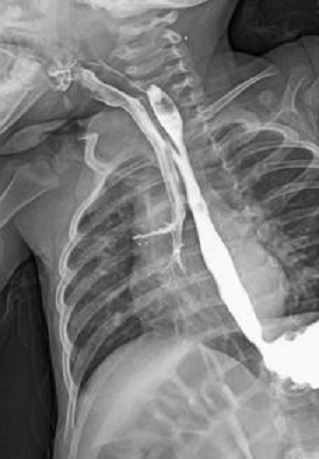
TOGD qui montre l'opacification de l'arbre trachéo-bronchique témoignant de la fistule œsotrachéal

**Figure 3:**
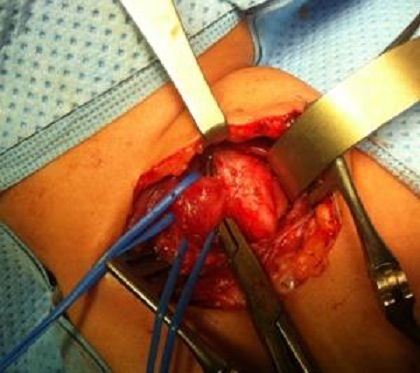
Aspect per-opératoire de la communication anormale entre l’œsophage et la trachée

**Figure 4:**
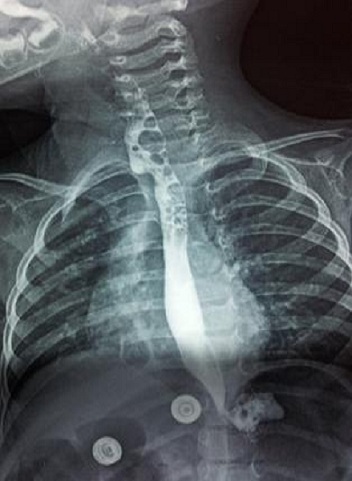
TOGD post opératoire de contrôle ne montrant pas de fistule oeso-trachéale

D.A, âgée de 21 mois, dans ses antécédents, on retrouve la notion de broncho-pneumopathies à répétition depuis la naissance avec des fausses routes et des accès de cyanose au moment des tétés. L'enfant a été bilanté dans une autre formation et y a bénéficié d'une gastrostomie puis nous a été référé pour complément de prise en charge. L'examen clinique trouve des râles ronflants à gauche, le cliché thoracique note un syndrome bronchique basal gauche. L'IRM thoracique a douté sur une communication oesotrachéale à hauteur de D2-D3 du côté droit. Un TOGD a objectivé une FOT (Figure 2). Une cervicotomie droite avec fermeture de la fistule (Figure 3) et interposition d'un lambeau du muscle omohyoïdien entre l'oesophage et la trachée ont été réalisés. Les suites post opératoires ont été simples avec un TOGD de contrôle qui est revenu tout à fait normal (Figure 4). Une fermeture de la gastrostomie a été réalisée 3 mois plus tard.

## Résultats

Dans cette série de 2 cas, l’âge moyen du diagnostic de FOT était de 17,5 mois. Cependant l'anamnèse a montré que la FOT était toujours symptomatique dès la naissance. Un TOGD a été réalisé pour les 2 patients et montrait une opacification trachéale. L'intervention a été réalisée dans les 2 cas par cervicotomie droite avec fermeture de la fistule et interposition d'un lambeau musculo-aponévrotique. L'alimentation orale a toujours été reprise après un contrôle radiologique de la suture digestive. Aucune complication à type d'encombrement bronchique persistant ou de fausses routes faisant craindre une récidive de la FOT n'a été notée chez les 2 patients.

## Discussion

La fistule oeso-trachéale est une anomalie rare représentée par un fin canal ascendant entre l'oesophage et la face postérieure de la trachée, à la hauteur du défilé cervico-thoracique [4].

Embryologiquement, vers la quatrième semaine, le diverticule respiratoire apparaît, puis le septum oesophagotrachéal sépare progressivement le primordium respiratoire en avant, et l'oesophage en arrière. Les anomalies de séparation de ces structures sont à l'origine des fistules oesotrachéales [5]. Son incidence serait de 1/50 000 à 1/80 000 naissances [6]. Des facteurs génétiques, environnementaux et tératogènes y seraient impliqués [5, 6].

Il existe une discrète prédominance masculine. Les malformations associées sont fréquentes, proches de celles qui sont observées dans les atrésies de l'oesophage. Elles entrent parfois dans le cadre du syndrome de VACTERL [1, 5].

La fistule empreinte un trajet ascendant d'arrière en avant justifiant ainsi l'appellation de fistule en H (bien qu'il s'agisse plutôt d'un N). La FOT est courte et son calibre est de quelques millimètres.

Le siège de la fistule est cervical bas dans 70 à 90% des cas se projetant entre C6 et D4 [7]. Les signes cliniques varient en fonction de l’âge auquel est fait le diagnostic et du calibre de la fistule mais ils sont évocateurs lorsqu'ils réunissent la triade de Helmsworth et Pryles associant toux et cyanose à la déglutition, ballonnement abdominal et pneumopathies à répétition. Cette triade par contre n'est pas constante, seuls la toux et la suffocation à la déglutition sont constamment rapportées et qui sont suffisantes pour évoquer le diagnostic de FOT. Le diagnostic est souvent retardé malgré les consultations précoces vu qu'on retrouve les mêmes symptômes dans les broncho-pneumopathies et le reflux gastro-oesophagien [1, 5, 8].

Le recours aux examens radiologiques et endoscopiques reste le moyen le plus approprié pour affirmer avec certitude la FOT ainsi que sa topographie. Le cliché sans préparation du thorax peut montrer, sur l'incidence de profil, une pneumatisation anormale de l'oesophage et des signes non spécifiques de pneumopathie. Une distension aérique de l'estomac et des premières anses grêles peut être présente sur le cliché d'abdomen sans préparation [1, 9].

La certitude diagnostique repose sur la visualisation directe de la fistule par opacification. Il est indispensable de réaliser une étude cinétique par radiocinéma plus performant que le transit oeso-gastro-duodénal. L'incidence de profil est la plus adéquate. Il ne faut pas hésiter à renouveler l'examen quelques jours plus tard, Beasley et Meyers rapportent une série de 30 fistules en H où ils ont fait le diagnostic dès le premier TOGD dans 73% des cas, et dans 100% des cas à la troisième tentative pour certains patients [1, 10].

La trachéoscopie est plus contributive au diagnostic que l'oesophagoscopie à cause des replis muqueux sur le versant oesophagien qui masque l'orifice de la fistule. Au cours de cet examen, on peut injecter du bleu de méthylène dans l'oesophage et voir s'il apparait dans la trachée.

Si la trachéoscopie est réalisée en préopératoire immédiat, il peut être utile de cathétériser la fistule par une sonde de Fogarty qui aidera à son repérage peropératoire [11]. D'autres méthodes, peu utilisées, ont été décrites telle la mesure de la pression partielle intragastrique en oxygène, le test de bullage, la tomodensitométrie et l'imagerie par résonnance magnétique.

Le traitement des FOT est toujours chirurgical dont le but est la suppression de la communication entre l'oesophage et la trachée. La date de l'intervention dépend de l’état respiratoire et nutritionnel de l'enfant.

La voie d'abord est une cervicotomie antérolatérale droite basse chez un patient correctement installé: décubitus dorsal, billot sous les épaules, tête tournée vers la gauche. L'axe trachéo-oesophagien est abordé en réclinant en dehors le muscle sterno-cléido-mastoïdien et le paquet jugulocarotidien. La dissection de l'espace intertrachéo-oesophagien conduit sur la fistule tout en faisant très attention aux nerfs récurrents. La fistule ainsi repérée est sectionnée au dépends de l'oesophage. Certains auteurs préconisent une interposition musculo-aponévrotique entre l'oesophage et la trachée afin de limiter le risque de récidive [1, 6].

La morbidité postopératoire se manifeste essentiellement par: la parésie récurrentielle généralement transitoire, trachéomalacie, atélectasies, reflux gastro-‘oesophagien et sténose de l'oesophage [6, 12].

La récidive de la fistule reste une complication rare. Cette morbidité est actuellement maitrisée grâce à la bonne préparation pulmonaire et nutritionnelle préopératoire et au choix correct de la voie d'abord qui évite la dissection excessive de la trachée et limite le risque de récidive de la fistule. A long terme, la guérison est obtenue sans séquelles [1, 5].

## Conclusion

Devant une symptomatologie respiratoire récidivante ou survenant notamment au cours de l'alimentation, il faut savoir évoquer la fistule oesotrachéale. Une fois suspectée, les examens complémentaires doivent la confirmer. Un traitement chirurgical précoce et bien conduit est la clé d'une guérison sans séquelles.
